# Complex myograph allows the examination of complex muscle contractions for the assessment of muscle force, shortening, velocity, and work *in vivo*

**DOI:** 10.1186/1475-925X-7-20

**Published:** 2008-07-10

**Authors:** Niels Rahe-Meyer, Matthias Pawlak, Christian Weilbach, Wilhelm Alexander Osthaus, Hainer Ruhschulte, Cristina Solomon, Siegfried Piepenbrock, Michael Winterhalter

**Affiliations:** 1 Department of Anaesthesiology, Hannover Medical School, Carl-Neuberg-Str. 1, D-30625, Hannover, Germany; 2 Department of Physiology, Biochemistry and Hygiene, University School of Physical Education, ul. Krolowej Jadwigi 27/39, Poznañ, Poland; 3 Institute of Physiology, University of Wuerzburg, Roentgenring 9, D-97070, Wuerzburg, Germany; 4 Department of Anaesthesiology, St. Josefs Stift Cloppenburg, Krankenhausstr. 13, D-49661, Cloppenburg, Germany

## Abstract

**Background:**

The devices used for *in vivo *examination of muscle contractions assess only pure force contractions and the so-called isokinetic contractions. In isokinetic experiments, the extremity and its muscle are artificially moved with constant velocity by the measuring device, while a tetanic contraction is induced in the muscle, either by electrical stimulation or by maximal voluntary activation. With these systems, experiments cannot be performed at pre-defined, constant muscle length, single contractions cannot be evaluated individually and the separate examination of the isometric and the isotonic components of single contractions is not possible.

**Methods:**

The myograph presented in our study has two newly developed technical units, i.e. a). a counterforce unit which can load the muscle with an adjustable, but constant force and b). a length-adjusting unit which allows for both the stretching and the contraction length to be infinitely adjustable independently of one another. The two units support the examination of complex types of contraction and store the counterforce and length-adjusting settings, so that these conditions may be accurately reapplied in later sessions.

**Results:**

The measurement examples presented show that the muscle can be brought to every possible pre-stretching length and that single isotonic or complex isometric-isotonic contractions may be performed at every length. The applied forces act during different phases of contraction, resulting into different pre- and after-loads that can be kept constant – uninfluenced by the contraction. Maximal values for force, shortening, velocity and work may be obtained for individual muscles. This offers the possibility to obtain information on the muscle status and to monitor its changes under non-invasive measurement conditions.

**Conclusion:**

With the Complex Myograph, the whole spectrum of a muscle's mechanical characteristics may be assessed.

## Background

The musculoskeletal system plays an important role both in the maintenance of one's vital functions and in the interaction with the outer world. The muscles' mechanical capacity is expressed by exerting force and shortening, and, most importantly, by combinations of force and shortening. The examination of these different aspects of muscular function *in vivo *in a clinical setting represents a methodological and technical challenge.

### Current technology for *in vivo *examination of muscle contraction

Most devices used for *in vivo *examination of muscle contraction are based exclusively on the assessment of pure force contractions [1-6]. Only a few *in vivo *devices allow examination of real shortening under controlled experimental isokinetic conditions [7-16], but none of these are used for routine clinical examination of the motor system diseases. In the isokinetic experiments, the extremity and its muscle are artificially moved with constant velocity by the measuring device and the resulting eccentric or concentric forces are recorded while a tetanic contraction is induced in the muscle, either by tetanic electrical stimulation or by maximal voluntary activation. Only tetanic or maximal voluntary contractions can be examined with these experimental procedures. The contractions have to be maintained over several seconds while the muscle length is decreasing from maximal to minimal in performing the movement, whereas both the pre-stretching length of the examined muscle and the number of actin-myosin bindings within the sarcomere are changing constantly. While the stretching length is changing, the tetanic cumulative contraction is induced, therefore the physical inertia of the system, the changing tenseness of the serial-elastic elements (e.g. tendons) and the fatigue of the muscle must be considered. All these variables make the physiological capacity of a single muscle impossible to quantify as a valid entity, independently of the special (isokinetic) experimental method. However, these diagnostic limitations prove unimportant when the isokinetic devices are used for training purposes [14-16].

### Aims of the study

The aim of the study was to perform a non-invasive *in vivo *evaluation of isotonic muscle functions at predefined muscle lengths, in order to characterize muscle force, shortening, velocity and work. For this purpose, we sought to develop a device with adjustable pre- and after-loads, whose exerting principle was independent of mass and friction, thus allowing for the load to remain constant during the phases of muscle contraction. The device also needed to offer the possibility of presetting the muscle length at the beginning and the end of the contraction. A device which would fulfill these two technical demands would support the assessment complex contractions that could be generated and examined at their optimal muscle length.

## Methods

Our previous publications referring to the complex myograph described the technical elements required for the measurement of isometric contractions (an axle-adjustment design, an extremity setting design, and a pre-stretching design) [17,18]. The present study introduces elements required for the assessment of isotonic contractions (a counter-force unit and a muscle length adjustment mechanism which allows for defining not only the pre-stretching length but also the maximal shortening length).

### Axle adjustment, extremity setting, stimulation and recording

**Figure 1:**
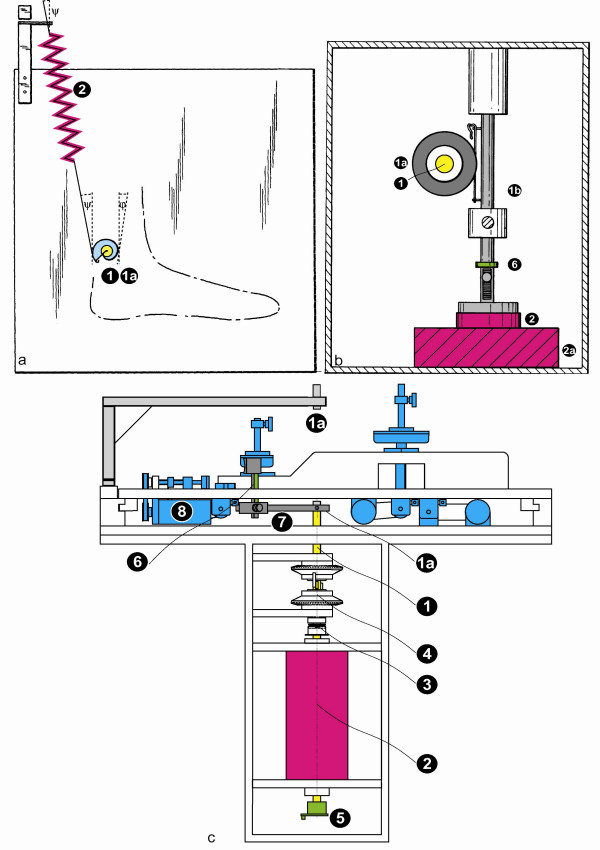
**a-c: Different counterforce units**. **1a **shows a side view on the spiral tension spring unit. A spiral tension spring (2) is attached to the rotational axle (1a) via a helical roller (1). The radius of the roller decreases (angle ϕ) when the spring is elongated. The spring does not touch the roller tangentially, but with a tracking error angle ψ: **1b **shows a side view on an electromagnetic plunger-coil unit. The rotating axle (1) is connected via a translating gear wheel (1a) to a linear axle (1b). Thus the coil (2) is inserted in the magnet core (2a). **1c **shows the brushless unipolar motor unit (2, magenta) in connection to the other elements of the device. With the help of laser beamers (1a, light gray), the subject's hand can be set onto the setting device (8, blue) and the thumb can be attached to the thumb lever (7, dark gray). A vertical axle (1, yellow) connects the thumb lever to the length-adjusting mechanism (4) and the motor (2). An adjustable friction clutch (3) prevents overstretching of the muscle. Sensors measure muscle length (5, green) and force (6, green).

The procedure of setting the extremity in the right position, of stimulating the *musculus adductor pollicis*, and of measuring force and muscle length is described in detail elsewhere [17]. The device features special axle adjustment and automatic setting which allow the adequate placement of the surface electrodes according to the morphology of the test subjects and a complete and constant transmission of the muscle vector to the sensors, independently of the muscle stretching length. The axle adjustment and setting values are stored and can be exactly reproduced in the following sessions. The muscle contractions are triggered indirectly via the *nervus ulnaris*, activated at the wrist's level by surface electrodes. In our experiments, electric pulses of a rectangular form of 0.1 ms were applied, with a frequency of one pulse every 100 seconds. The amplitude of the electric pulses – between 10 mA and 50 mA – was evaluated for each subject in a pre-test. This level was 15% higher than the one necessary to obtain maximum amplitude of the mechanical peak twitch contraction and was therefore called the supra-maximum pulse. The pre-stretching and contraction forces o were measured at the thumb's level with a beam-measuring gauge (see 6 in Fig. 1c). The muscle's ideal lengths were determined via a potentiometer (see 5 in Fig. 1c) on the apparatus axle (see 1 in Fig. 1c) [17].

### Development of a counterforce unit

Muscle contractions act via the bone levers of the extremity perpendicularly on the physiological axis and can thus be transferred onto the axle of the measuring device. Experimental forces or loads can be similarly transferred via the axle of the device onto the muscle. These loads must remain constant during contractions, although they change the rotation angle, velocity and acceleration. Simple weights are inappropriate because of additional inertial forces that appear during acceleration. Three technical solutions are described, applying adjustable constant forces.

Fig. 1a. shows a simple, exclusively mechanical embodiment of the counterforce unit that can produce constant torques for the stretching or against the contraction of the muscle. A spiral tension spring (2) is attached to the rotational axle (1a) via a helical roller (1). In cross section, the helical roller takes the shape of a curve according to the following formula:

r(ϕ) = r_0_(1+ ϕ 2r_0_/x_0_)^-1/2^

with the tracking error angle ψ:

cos ψ = (1+ ψ 2r_0_/x_0_) [(1+ ϕ 2r_0_/x_0_)^2 ^+ (r_0_/x_0_)^2^]^-1/2^

where ϕ is the angle of rotation, r_0 _is the initial radius, r(ϕ) is the radius after the rotation of the roller with the angle ϕ and x_0 _is the initial pre-lengthening of the spiral tension spring. When the muscle contracts, the axle and the roller rotate and the spring is elongated. The increasing tensile force is compensated by the decreasing radius of the roller and a constant torque is achieved. The spring, for which a tensile force is selected as needed, can be exchanged with others of greater or lesser tension force as long as they have the same initial pre-elongation. Thus, different loads can operate on the rotating axle and remain constant during the muscle contraction. [19]

Fig. 1b shows an electro-magnetic counterforce unit. The rotating axle (1) is connected via a translating gear wheel (1a) to a linear axle (1b), so that the rotation is transformed into a longitudinal movement. At the bottom end of the linear axle, an electromagnetic coil (2) is inserted in, and respectively penetrates a magnet core (2a). Thus, the electromagnetic force can be adjusted by the current through the coil and it remains constant as long as the same number of windings of the coil's wire remains in the magnetic gap. [19]

A third technical solution is to assemble an electro-magnetic motor directly onto the rotation axis (1, see Fig. 1c). A standard electric motor, however, is inappropriate, because its electromagnetic field is not homogeneous and therefore creates torque ripple or fluctuations in force; moreover, oscillations may follow. To prevent this, we used a brushless unipolar motor with a homogenous field, which resulted in a practically constant resultant torque independent of the rotor angle (2) [20-24]. The maximum force or pre-load of the counterforce unit is 60 Newton. To prevent overstretching of the muscle, an adjustable friction clutch (8) is installed in the axle [20,21].

### Development of a muscle length-adjusting mechanism

**Figure 2:**
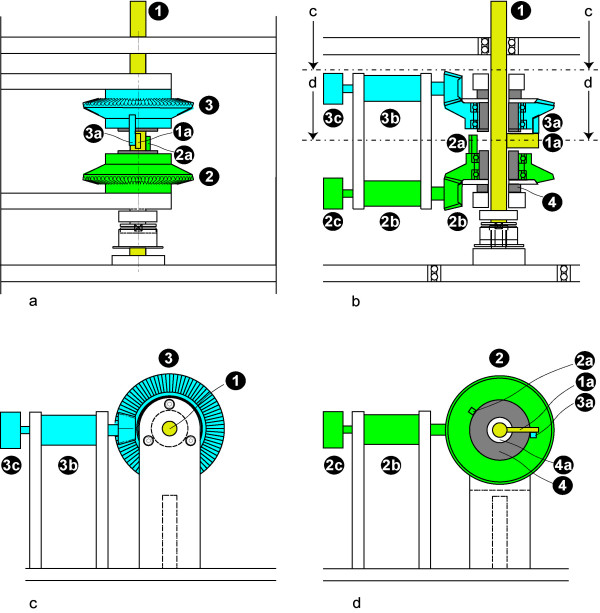
**a-d: Length-adjustment mechanism**. **2a **shows the length-adjustment mechanism in detailed side view (see also Fig. 1c), **2b **shows a 90 degree rotated side view section, longitudinally cut through the axle, **2c **and **2d **show transversal sections as viewed from above in **2b**. The stopping mechanism for shortening consists of a stopping bolt (2 a) which can be rotated via an adjustment wheel (2) by an adjustment motor (2b). The position of the bolt is measured by a potentiometer (2c). The pre-stretching mechanism (3, 3a, 3b, 3c) can be independently adjusted. The adjustment wheels rest via bearings on two parts of a guiding tube (4) so that there is an air gap (4a) that prevents any direct contact between the adjustment wheels and the axle (1). A third bolt (1a), which moves parallel to the thumb lever (see 3 in Fig. 1a), is mounted on the axle.

A length adjustment mechanism was developed (see Fig. 2a–d), which allows not only to define the pre-stretching length [17] but also to limit the shorting of the muscle during the contraction. The length adjustment mechanism is set around the axle of the apparatus without contacting it (see 4, 4a, 1). A shortening stopping bolt (3a) and a pre-stretching stopping bolt (2a) can be adjusted independently of one another by means of adjustment wheels (3, 2) and adjustment motors (3b, 2b). The adjustment wheels rest via bearings on two parts of a guiding tube (4), creating an air gap (4a); this prevents any direct contact between the adjustment wheels and the axle (1). On the axle, a third bolt (1a) – which moves parallel to the thumb lever (see 3 in Fig. 1a) – is mounted. The adjustment range of axle rotation – which allows different muscle lengths – is limited by both the distance between the axle bolt (1a) and the shortening stopping bolt (3a) on the one hand and by the distance between the axle bolt (1a) and the pre-stretching stopping bolt on the other hand (2a).

By adjusting the shortening stopping bolt, (3a) the muscle's shortening can be limited, so that pure isometric or complex isotonic-isometric contractions (end-stop contractions) can be performed. By adjusting the pre-stretching stopping bolt (2a), the muscle's pre-stretching can be limited, so that pure isotonic or complex isometric-isotonic afterload contractions can be performed. Each position of the bolts is registered by electrical measurement recorders (3c, 2c). This information is stored and the mechanism can be moved automatically into position for the same subject at a later time point [17,20].

### Protocol of the experiment for rest-stretching and isotonic maxima curve (Experiment 1)

The extremity of the test subject was properly set onto the device. The stimulation electrodes were placed on the skin above the *nervus ulnaris *at wrist level. With sub-maximal test pulses, the position of the electrodes was optimized. In a first pre-test, the amplitude of the necessary supra-maximal stimulus was determined by applying increasing electric pulses. In a second pre-test, the maximum stretching-force was determined by applying incremental stretching forces.

As a preparation for Experiment 1, the shortening stopping bolt and the pre-stretching stopping bolt of the muscle length adjustment mechanism were positioned in a manner that prevented them from limiting the space for pre-stretching or shortening.

Experiment 1 consisted of 10 consecutive experiment units, each using a different stretching force applied by the counterforce unit. At the beginning of each experiment unit, the muscle was stretched for approximately five seconds, which guaranteed the completion of the stretching process. A supra-maximal stimulus led to a length contraction after which the stretching force returned to zero. Stretching lengths and forces and the contraction shortening were recorded [25].

### Protocol of the experiment for isotonic afterload contractions with velocity-length relation (Hill) and work-length relation (Experiment 2)

The extremity of the test subject was properly set onto the device. The stimulation electrodes were placed on the skin above the *nervus ulnaris *at wrist level. With sub-maximal test pulses, the position of the electrodes was optimized. In a first pre-test, the amplitude of the necessary supra-maximal stimulus was determined by applying increasing electric pulses.

As a preparation for Experiment 2, the pre-stretching stopping bolt was positioned at the muscle length to be examined – e.g. the optimum length for shortening or work – and the shortening bolt was positioned so that it did not limit the space for shortening. As a rule, the maximum counter-force applied by the counter-force unit should be slightly higher than the maximum contraction force which can be expected at a given muscle length. Besides, the minimum counter force should be the necessary pre-stretching force for that given muscle length. This information should be obtained from previous testing e.g. see above "isotonic muscle contraction".

Experiment 2 consisted of 11 consecutive experiment units, each using a different stretching force applied by the counterforce unit. After 18 ms of applying counterforce, a complex isometric-isotonic contraction induced by a supra-maximal stimulus occurs, after which the counter force returns to the minimum value. The stretching lengths and forces and the contraction shortenings and forces were recorded [25].

## Results

### Measurement of rest-stretching and isotonic maxima curve (Experiment 1)

**Figure 3:**
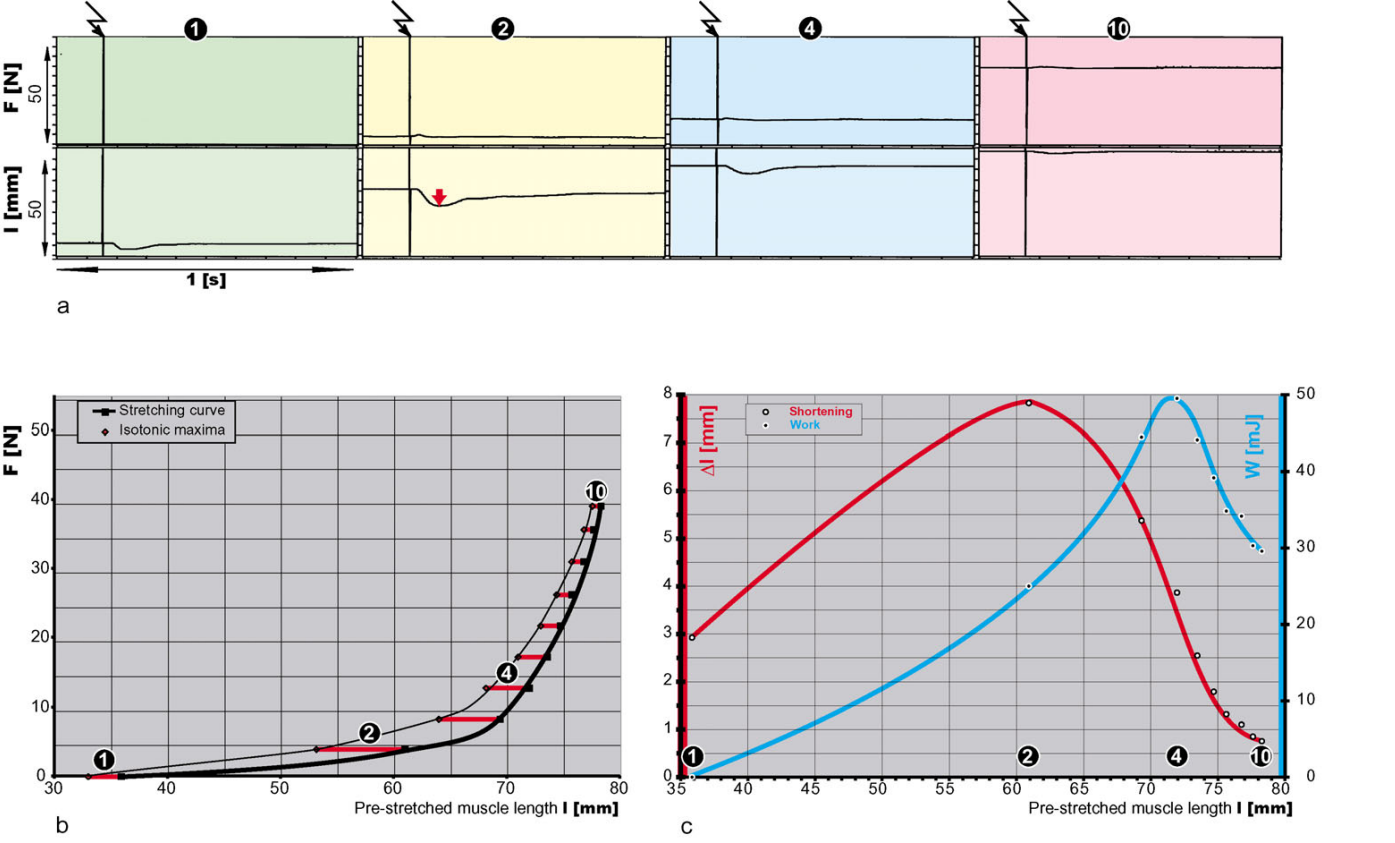
**a-b: Isotonic contractions: optimum shortening and optimum work in relation to stretching length**. Fig. 3a shows four experiment units (1, 2, 4, 10) from a sequence of ten units with rising stretching forces (top fields) and stretching lengths (bottom field). With each stretching force and length of the muscle, a single supra-maximal stimulus was applied for 0.1 ms (vertical line with zigzag arrow), achieving an isotonic shortening that reached its highest value in experiment unit 2 (see red arrow). In the graph Fig. 3b, the stretching curve is shown, with isotonic length contractions plotted as red lines for each measuring point. In the graph Fig. 3c the shortening in relation to muscle length (red line), and the contraction work in relation to muscle lengths (blue line) are shown.

Fig. 3a shows four experiment units (1, 2, 4, 10) from a sequence of ten units. The top fields illustrate experiment unit 1, where no force was applied, in contrast to experiment unit 10, where a stretching force of 40 N was applied. The bottom fields illustrate the muscle lengths. The stretching lengths increase with the stretching forces, starting from 36 mm in experiment unit 1 and ending at a stretching length of 78 mm in experiment unit 10. For each stretching force and length of the muscle, a single supra-maximum stimulus was applied for 0.1 ms (vertical line with zigzag arrow), achieving a standardized contraction in which all muscle fibres were activated simultaneously. The bottom fields illustrate how these isotonic shortenings could be observed after a small latency period. As experiment unit 1 demonstrated, the shortenings were small at low muscle-stretching lengths, reached a peak at longer muscle-stretching lengths (see red arrow) and then decreased at the longest muscle-stretching lengths (experiment units 4 and 10). Graph 3b illustrates the stretching curve. The isotonic length contractions are plotted as red lines for each measuring point. Graph 3c represents the shortenings in relation to muscle lengths (Fig. 3c), where the muscle's typical optimum stretching length for shortening (approximately 61 mm) can be determined. In the graph representing the contraction work in relation to muscle lengths (Fig. 3c), the muscle's typical optimum stretching length for work (approximately 72 mm) can be determined.

### Measurement of isotonic afterload contractions with velocity-length relation (Hill) and work-length relation (Experiment 2)

**Figure 4:**
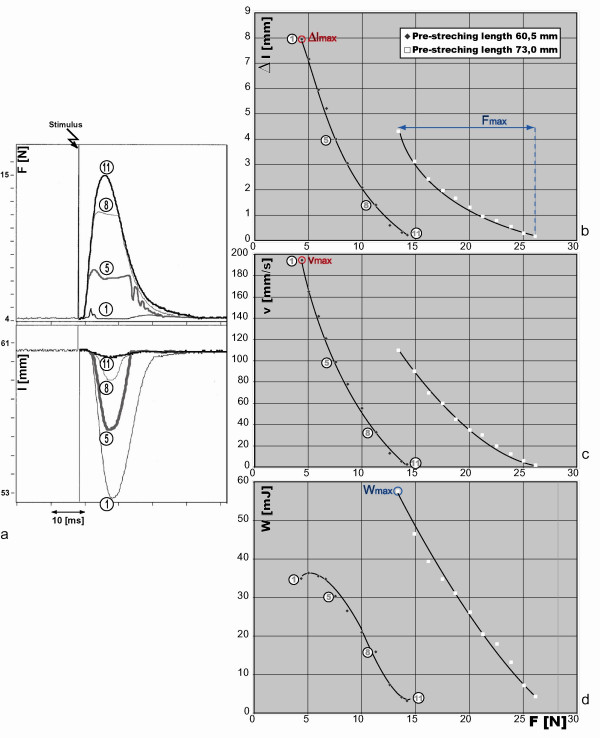
**a-d: Isotonic afterload contractions: relation between shortening, velocity, work and stretching force**. Fig. 4a shows four experiment units (1, 5, 8, 11) from a sequence of eleven units with complex isometric-isotonic contractions. The top field shows the contraction forces and the bottom field the shortenings. The vertical line with zigzag arrow indicates the timing of the electric stimulus. High contraction force leads to small shortening and vice versa. Fig. 4b shows the shortenings (Δl), Fig. 4c shows the shortening velocities (v) (time derivative of the shortening) and Fig. 4d shows the work W = F_*_Δl, where Δl equals the shortening and F the counterforce. Graphs of two different pre-stretching lengths are shown.

Fig. 4a shows four experiment units (1, 5, 8, 11) from a sequence of 11 units at a muscle length of 56 mm. The top field shows the forces and the bottom field indicates the lengths. After 18 ms of applying counterforce, a single supra-maximum stimulus was applied for 0.1 ms (vertical line with zigzag arrow), achieving a standardized contraction in which all muscle fibres were activated simultaneously. After a short latency period, the contraction induces force development until the counterforce (= after-load) is reached. In experiment unit 1, the counterforce is just the necessary pre-stretching force (3 N = pre-load), in experiment units 2–10 the counterforce level is reached by the contraction, an isotonic shortening of the muscle begins, and in experiment unit 11 the counterforce (11 N) is higher than the maximal isometric contraction. The higher the counterforce, the lower are the values reached by the shortenings amplitudes.

In the graphs (Fig. 4b–d) the results of two experiments at two prestretching lengths are shown: at 60.5 mm muscle length for optimum shortening and at 73 mm for optimum force or work. Fig. 4b shows the shortenings (Δl), Fig. 4c shows the shortening velocities (v) (time derivative of the shortening) and Fig. 4d shows the work W = F_*_Δl, where Δl equals the shortening and F the contraction force – all in relation to the contraction forces (F). This figure demonstrates that the assessment of the maximal force, shortening, velocity, and work of a particular muscle was possible [25].

## Discussion

The present study presents a measuring device that works with complex supra-maximal contractions. The length adjusting mechanism used allows for the stretching length and the shortening length to be determined separately. The counterforce unit produces adjustable pre- and after-loads which remain constant, no matter what muscle shortenings, velocities or accelerations are applied. Data on isotonic and mixed isometric-isotonic contractions of the human *musculus adductor pollicis in vivo *has been previously presented. From these data, the individual maximal values for force, shortening, velocity, and work may be obtained.

The current technical developments allow only for *in vivo *isometric contractions to be assessed, even at optimal stretching level [17,18]. Regarding the clinical setting, however, there is no satisfactory solution to investigate muscle properties *in vivo *other than isometric contractions. The importance of *in vivo *measurements of non-isometric muscle functions cannot be fully determined until new diagnostic devices will support the collection of additional data in different clinical areas. Age, sex, fatigue, disease, medication and training are known to distinctively affect different types of muscle fibres. These factors influence the isometric properties of the muscle differently than the isotonic properties. These findings were based not only on *in vitro *studies on animal muscle fibres [26-29], but also on human single muscle fibres [30,31]. The state of the dynamic/isotonic muscle functions plays a more important role than that of static muscle force in everyday life or in the course of a neuromuscular disease [32,33]. Designing a diagnostic tool for dynamic muscle function is necessary for both medical research and clinical routine diagnostic. With the Complex Myograph this relevant biological-medical problem could be solved, and *in vivo *non-invasive measurements of both isometric and isotonic muscle functions can be performed at the corresponding optimal muscle length.

The three technical solutions for a counter-force unit, described in our study, work with adjustable axial forces which remain constant during the experimental procedure. The first mechanical solution (Fig. 1a) is robust and simple in service and maintenance. Even though it only allows a gradual variation of counterforces, its advantage lies in is the simplicity and transparency of the experimental design. The electro-magnetic solution (Fig. 1b) is also simple, since it uses prefabricated components that are easy to obtain and assemble i.e. a coil and a magnet core. This solution allows an unrestricted, but maximum force limited variation of counter-forces due to the capacity of the coil and to the delicate bearings that transform the rotating movement into a linear movement which is centred over the magnetic gap.

The Hall-motor solution (Fig. 1c) is very robust and can support high counter-forces. It operates directly on the rotating axle and has only a few movable elements which all can rotate in horizontal planes so that there is no interference with gravitation. However, the necessity to assemble the motor by hand may constitute a limitation for its application.

The first two solutions (Fig. 1b + c) have been used for the education of biomedical students – the application of an expensive, specially designed unipolar motor (Fig. 2) could not be accomplished in this field. A predecessor model of the third solution (Fig. 2) has already been used for the examination of patients.

With the length-adjusting mechanism (Fig. 2), the pre-stretching length of the muscle can be defined [17] and the shortening can be limited without interfering with the rotation of the axle and with the muscle contraction, respectively. Using the counterforce and the length-adjusting mechanism complex, isometric-isotonic contractions can be produced at every possible muscle length. The settings of these mechanisms can be stored and can be used again for the same subject at a later point in time.

The measurements performed during the study (Fig. 3, Fig. 4) show that the planned experimental procedures could be accomplished with the technical developments presented above. The muscle could be examined at different stretching lengths, while optimal lengths for shortening, force or work could be defined. At these optimal lengths, the properties of an individual muscle may be assessed, not only in terms of maximal force, but also as maximal shortening, velocity, and work. Velocity characterizes a muscle better than shortening does, because it is nearly independent of the muscle length or the number of sarcomeres which are lined up in a muscle. The functional curve (Fig. 4c) shows a typical hyperbolic form [34,35]. The muscle work (W) is calculated as the product of afterload (F) and shortening (Δl) (W = F_*_Δl). The muscle length at which the maximal work and maximal force can be assessed is the length with the most actin-myosin bindings. Nevertheless, the optimum stretching length for shortening contractions and for velocity is smaller and with less actin-myosin bindings. This is because in isotonic contractions the preload (stretching force) necessary to stretch the muscle works before the contraction and has to be compensated for during the contraction as a part of the afterload, hence diminishing shortening and velocity.

### Special limitation

During the isotonic contractions (Fig. 3a), the stretching forces in the top fields show some minor fluctuations. This is also the case in the isotonic afterload contractions (Fig. 4a). In the top field the force plateaus are not free from fluctuations either, because we did not use a Hall-motor with an ideal constant characteristic line in our measurements but a much simpler solution with a rotary magnet.

### General limitations

The experiments were performed exclusively with single twitches. The isometric single twitch represents the golden standard for the evaluation of muscle function in many clinical areas, e.g. in the assessment of the effect of neuromuscular blocking drugs during anaesthesia. Every single twitch represents a defined contraction unit, because in a single twitch all myosin filaments within the muscle are activated during a predefined, constant period of time. A single twitch measurement is simple and quick. Single twitch stimulations are not painful in contrast to tetanic stimulations. Moreover, they have a better repeatability than voluntary contractions and do not lead to muscle fatigue. Our development added another important advantage: the possibility to assess muscle function by using single twitches at predefined muscle lengths. The single twitches have a limited duration, which leads to a limited contraction length that allows for the number of actin-myosin bindings to remain relatively constant during the contraction.

On the other hand, in single twitches, the active state (the actin-myosin interaction) is maximal only for a short period of time, which is too short to completely tense the serial elastic elements. Thus, the elastic elements are continuously tensed during the active state by using up some of the contraction energy. Only the rest of the energy will therefore be used for contraction on the extremity lever. At the end of the active state, the energy used on the elastic elements is released and contributes to the prolongation of the effect on the extremity lever.

Only in the tetanic contractions the duration of the active state is so long that the serial elastic elements (tendons) can be completely tensed and lengthened in the first phase of the contraction. From then on, the tendons undergo no additional changes, so that the actin-myosin interaction may be transferred onto the extremity lever without loss of contraction energy [7]. However, due to methodical reasons, isotonic tetanic contractions cannot be soundly related to defined muscle stretching length. Shortening obtained with tetanic contractions does not offer conclusive information because this type of contraction is not time-limited and ends independently from the starting muscle length when the maximal shortening is reached. In addition, muscle velocity obtained with tetanic contractions *in vivo *cannot be related to an optimum muscle length because either the muscle stretching is too restricted by the joint to define an optimum length or the number of actin-myosin bindings changes during the acceleration phase of the contraction. Hence such measurements as those described in Fig. 3 or Fig. 4 are only possible with single twitches. The solution to this problem is to assess isometric and isotonic muscle function with single twitches at the respective optimum muscle lengths and every once in a while to compare the isometric single twitch with the isometric tetanic contraction, so that the component of single twitch invested in the tension of the serial elastic elements may be ascertained. Tetanic contractions, which were not the subject of our study, may also be evaluated with our device [24].

## Conclusion

Statical muscle force is only one aspect of the muscle functions. For the clinical activity, the dynamic muscle functions are of higher importance, since they are particularly influenced by muscle diseases and by therapy. Complex Myograph supports an extensive, non-invasive evaluation of these muscle functions in patients.

Single supramaximal contractions can be produced in the form of isotonic or even complex isometric-isotonic contractions. The starting (stretching) length and the maximal shortening length can be predefined separately. A sophisticated counterforce unit can set the muscle under infinitely adjustable pre- and after-loads which remain constant during shortening, changing velocities and contraction acceleration. On the stretching curve, the optimum length for shortenings and the optimum length for work can be recorded, based on which the individual physiological properties of a muscle can be assessed and maximal values for force, shortening, velocity, and work can be quantified. The possibility to evaluate directly dynamic muscle functions opens new medical-biological perspectives.

## Competing interests

NRM developed the device and is the owner of two international patents. The authors declare that they have no other competing interests.

## Authors' contributions

NRM developed the device and wrote the manuscript, NRM, MW, MP, HR and WAO carried out the experiments on different generations of laboratory devices, MW and SP coordinated the experiments, CW and CS helped with the physiological interpretation of the data and with preparing the manuscript's draft.
